# Real-time monitoring of the physiological state of CHO cells in bioreactor by using a dual spectroscopic strategy

**DOI:** 10.1186/1753-6561-9-S9-P48

**Published:** 2015-12-14

**Authors:** Franck Courtès, Bruno Ebel, Emmanuel Guedon, Annie Marc

**Affiliations:** 1 Laboratoire Réaction et Génie des Procédés, UMR 7274, CNRS-Université de Lorraine, 2 avenue Forêt de Haye, TSA 40602, 54518 Vandœuvre-lès-Nancy Cedex, France

## Background

Bioprocesses of mammalian cell culture have become essential in pharmaceutical fields for the production of recombinant therapeutic proteins such as monoclonal antibodies or for tissue therapy. Following the FDA's recommendation to apply Process Analytical Technology (PAT) approach as the mean to control production processes and ensure the quality of the end-product for patients, Near Infra-Red (NIR)and dielectric spectroscopic technologies have gained great attention over the past decade as online tools for cellular bioprocesses monitoring [1,2]. In such processes, cells are one of the most critical parameter, because their physiological state directly impacts the final product titer through their productivity as well as the product quality. Animal cells are typically classified into viable or dead entities via Trypan Blue exclusion technique, but lysed cells are not accounted for, although they could represent a significant proportion of the cell population in bioreactors. In addition to reducing productivity, cell lysis causes the release of intracellular proteases and glycosidases, which can degrade secreted recombinant protein of interest. Therefore, they should be considered for optimal control of processes. In this work, we propose the first online strategy combining NIR and dielectric spectroscopies for in-depth characterization of cellular growth and physiology of CHO (Chinese Hamster Ovary) cells cultivated in bioreactors.

## Materials and methods

CHO 320 cell line was cultivated in 2 L bench-top bioreactors regulated at 90 rpm, 50% air saturation, pH 7.2 and 37 °C for the standard culture conditions. Culture medium BDM was a mix 5:5:1 vol. ratio of IMDM, Ham F12 and NCTC media respectively, supplemented with 3 mM of glutamine. In order to have robust calibration model, various operating conditions (glucose and/or glutamine feeding and temperature shift at 32 °C) have been applied. During cultures, viable and dead cell densities were determined offline by Trypan Blue dye exclusion staining. The number of lysed cells was calculated based on the extracellular activity of lactate dehydrogenase [3]. A sterilizable NIR probe served for spectra acquisition of medium molecules vibrations due to photon absorption between 4,000 and 10,000 cm-1. The region of interest for modeling was chosen between 5,600 to 10,000 cm−1of NIR spectra with a 8cm-1 resolution that were pre-treated with first derivative and SNV (standard normal variate).PLS (partial least-squares) modeling with venetian blinds cross-validation was adopted for the quantitative regression model. NIPALS algorithm was used to perform PLS regression. A dielectric probe (Fogale Biomass System) was used concomitantly to measure the overall permittivity and conductivity of cultures submitted to an alternating electric field.

## Results

Combining both spectroscopic probes and chemometrics data analysis, we were able to follow on-line three key parameters: viable cells density (VCD), dead cells density (DCD) and indirectly lysed cells density (LCD) via LDH (lactate dehydrogenase) activity. The parameters employed to build the different models of calibration and the statistic quality of these models are summarized in Table 1.

**Table 1:** Parameters of model calibration and prediction.

	Viable cell density	Dead cell density	LDH activity
n_calibration_ / n_prediction_	83 / 13	61 / 12	48 / 13
Regression model	Linear	Exponential	PLS
Calibration range	2 × 10^5 ^- 6 × 10^6 ^cells.ml^-1^	1 × 10^4 ^- 1 × 10^6 ^cells.ml^-1^	10 - 600 U.l^-1^
Preprocessing	N.A.	N.A.	1^st ^derivative, SNV
Latent factor	N.A.	N.A.	7
R²	0.94	0.97	0.94
RMSE	4.4x10^5 ^cells.ml^-1^	3.2x10^5 ^cells.ml^-1^	N.A.
RMSEC	N.A.	N.A.	29 U.l^-1^
RMSECV	N.A.	N.A.	44 U.l^-1^
RMSEP	1.25x10^5 ^cells.ml^-1^	7.4x10^4 ^cells.ml^-1^	9 U.l^-1^

NIR spectroscopy calibration models were generated for LCD through on-line monitoring of extracellularly released LDH upon cell membrane disruption. NIR spectrometry can successfully monitor LDH activity in culture medium online. Knowing that there are 110 units of LDH x10^-9 ^cells (data not shown), the total density of dead cells (TDCD) having released their pool of LDH in the culture medium, can be estimated. LCD is determined as: LCD = TDCD - DCD. In parallel, on-line measurements *via *dielectric spectroscopy of permittivity was confirmed to be a good monitoring parameter for VCD with R^2 ^greater than 0.9, while conductivity proved to be an innovative and effective way to monitor DCD (R^2^> 0.9) potentially resulting from a gradual alteration of membrane permeability.

**Figure 1:**
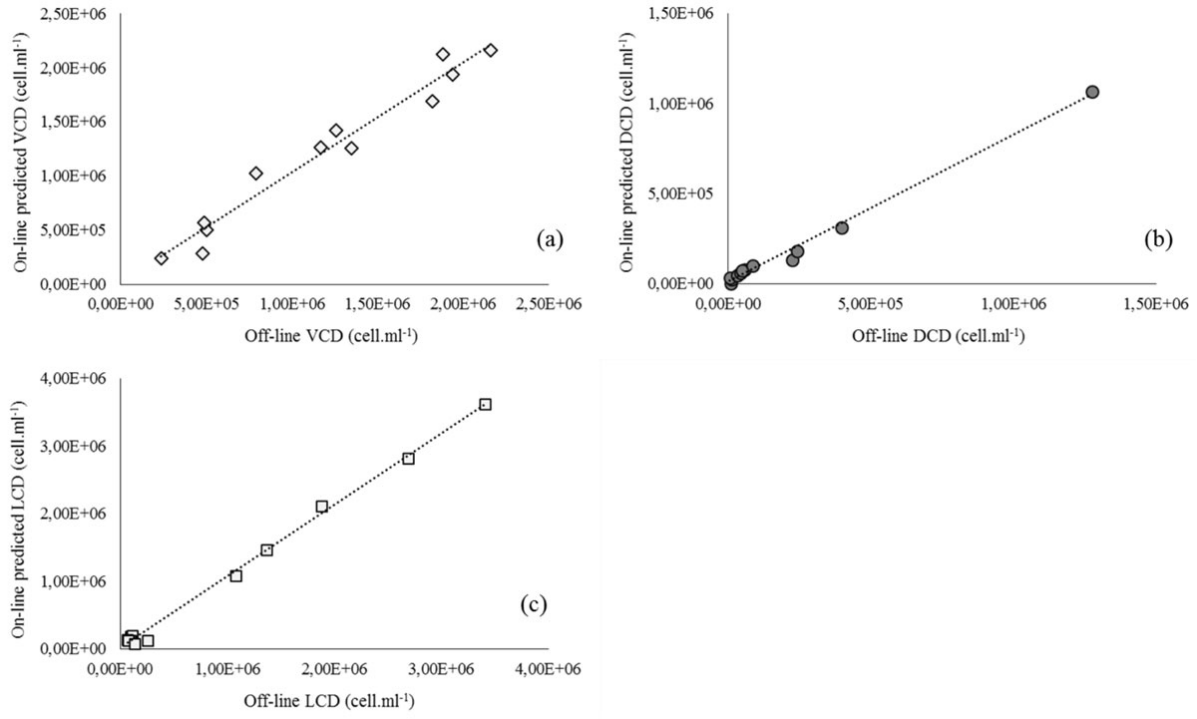
**Off-line (experimental)and predicted values of (a)viable cells density (VCD,●), (b) dead cells density (DCD,◇) and (c) lysed cells density (LCD,□)**.

Thus, the combined use of NIR and di-electric spectroscopies allowed to determine in real time throughout culture the densities of the different cellular sub-populations: viable cells (VCD), dead cells with permeable membranes (DCD) and lysed cells (LCD) (Figure 1).

It is interesting to note that starting from the stationary growth phase (after day 3) corresponding to 1.9 × 10^6 ^viable cells.mL^-1^, the lysed cell population accounts for a significant proportion of the total cell density. On the basis of real time monitoring, strategies could be developed to minimize the onset of cell lysis, which could increase process yield.

## Conclusions

This work presents the first use of on-line NIR monitoring of extracellular LDH activity for the real-time determination of lysed cell density (LCD) as well as the first evidence that DCD can be monitored online through conductivity reading. It proposes a successful combined use of NIR and dielectric spectroscopies for in-depth on-line characterization of cellular population densities in real-time. These results demonstrate the strong potential for a dual spectroscopy strategy to enhance real-time monitoring towards better understanding and control of bioprocesses. In particular, it will allow identifying armful culture conditions that could result in direct cell lysis. Such knowledge will improve understanding, control as well as performance of bioprocess.

## Acknowledgements

This work was financially supported by the French National Research Agency (ANR) through SPECTRE project.
